# Frontline polatuzumab vedotin for diffuse large B‐cell lymphoma: A survey of clinician impressions

**DOI:** 10.1002/jha2.505

**Published:** 2022-06-16

**Authors:** Ajay Major, Edward R. Scheffer Cliff, Daniel A. Ermann, Urshila Durani, David A. Russler‐Germain

**Affiliations:** ^1^ Section of Hematology/Oncology, Department of Medicine University of Chicago Chicago Illinois USA; ^2^ Department of Health Policy and Management Harvard T.H. Chan School of Public Health Boston Massachusetts USA; ^3^ Program on Regulation, Therapeutics and Law Brigham and Women's Hospital, Harvard Medical School Boston Massachusetts USA; ^4^ Department of Hematology/Oncology, Huntsman Cancer Institute University of Utah Salt Lake City Utah USA; ^5^ Division of Hematology, Department of Medicine Mayo Clinic Rochester Minnesota USA; ^6^ Division of Oncology, Department of Medicine Washington University School of Medicine St. Louis Missouri USA

**Keywords:** chemotherapy, clinical trials, lymphomas

## Abstract

In the POLARIX trial, pola‐R‐CHP demonstrated improved progression‐free survival (PFS) compared to R‐CHOP in untreated intermediate‐ to high‐risk DLBCL. We surveyed practicing clinicians regarding their interpretation of POLARIX, including impressions of efficacy, safety, and cost. Of 174 respondents, most from academic centers (82%) in the United States (57%), 70% stated they would not replace R‐CHOP with pola‐R‐CHP due to insufficient PFS difference, lack of overall survival benefit, and excessive cost. Respondents not recommending pola‐R‐CHP expressed concerns about financial implications for both society and patients. We observed considerable heterogeneity in both study interpretation and plans for real‐world implementation of pola‐R‐CHP.

1

Treatment of newly diagnosed diffuse large B‐cell lymphoma (DLBCL) has remained largely unchanged for 20 years since rituximab was added to the backbone of cyclophosphamide, doxorubicin, vincristine, and prednisone (R‐CHOP) [1]. Several randomized controlled trials have sought to improve upon R‐CHOP [2], including intensified chemotherapy, post‐R‐CHOP maintenance, and novel therapies added to R‐CHOP [3]. Most of these trials did not meet their endpoint of improved progression‐free survival (PFS), and none became standard‐of‐care [4]. Most recently, the POLARIX study [5], an international, double‐blind, placebo‐controlled randomized phase 3 trial, evaluated pola‐R‐CHP (replacing vincristine in R‐CHOP with the anti‐CD79b antibody–drug conjugate polatuzumab vedotin) versus R‐CHOP. It enrolled adults with untreated intermediate‐/high‐risk DLBCL (defined by International Prognostic Index [IPI] score ≥2), with PFS as its primary endpoint and overall survival (OS) and safety as secondary endpoints. POLARIX met its primary endpoint of improved PFS, with no new safety signals and immature OS data, when the findings were presented at the American Society of Hematology Annual Meeting and simultaneously published in December 2021. Given the potential for pola‐R‐CHP to replace R‐CHOP as standard frontline treatment for DLBCL, we conducted a survey of practicing clinicians to understand their real‐world interpretation of the POLARIX results, including impressions of the efficacy, safety, and cost of pola‐R‐CHP.

We developed an electronic survey that included clinician demographics, DLBCL practice patterns, a discrete‐choice experiment (DCE) comparing R‐CHOP with a hypothetical regimen “S‐FLOP,” and perceptions regarding pola‐R‐CHP. The survey was open from January 11 to February 20, 2022, and distributed via email and social media to academic medical centers, private practice groups, and lymphoma‐focused professional societies. The survey, available in Supporting Information, was approved by Washington University in St. Louis Human Research Protection Office. Free‐text responses were qualitatively coded in consensus by two authors.

Of 302 subjects who opened the survey, 174 (58%) consented to participation and had response data available. The majority of respondents worked in an academic health system (82%), were located in the United States (US, 57%), were medical doctorates (69%), and identified as hematologists (42%) or hematologist–oncologists (35%) (Table 1). Respondents, nearly half of whom were involved in DLBCL clinical trials, had a median of 10 years of clinical experience treating patients with lymphoma and saw a median of five patients with DLBCL per week. Regarding contemporary practice patterns, most respondents used intensified therapy for double‐/triple‐hit lymphoma (67%), and many utilized high‐dose methotrexate for central nervous system (CNS) prophylaxis in patients with high CNS‐IPI scores (47%). Most respondents had a mix of good and poor clinical experiences with polatuzumab vedotin in the relapsed DLBCL setting (48%).

**TABLE 1:** Demographics and diffuse large B‐cell lymphoma (DLBCL) practice patterns of survey respondents

Survey question	Total (*N* = 174)	%
Which best describes the healthcare setting in which you work? (*N* = 170)		
Academic health system	139	82%
Private practice	8	5%
Hybrid model (private with academic affiliation)	18	11%
Other	5	3%
In what country are you based? (*N* = 150)		
USA	86	57%
Spain	13	9%
UK	13	9%
Australia	12	8%
Canada	4	3%
India	4	3%
Saudi Arabia	3	2%
Czechia	2	1%
France	2	1%
Switzerland	2	1%
Brazil	1	1%
Chile	1	1%
Germany	1	1%
Iraq	1	1%
Ireland	1	1%
Israel	1	1%
New Zealand	1	1%
Sweden	1	1%
Thailand	1	1%
What is your degree? (*N* = 172)		
MD, DO, MBBS, MBChB, or equivalent	118	69%
MD and PhD	40	23%
PharmD or other pharmacy degree	9	5%
NP (i.e., APN, DNP, or equivalent)	3	2%
PA	1	1%
Other	1	1%
What type of clinician are you? (*N* = 172)		
Hematologist	72	42%
Hematologist/medical oncologist	61	35%
Hematology, oncology, or hematology/oncology fellow	15	9%
Medical oncologist	13	8%
Pharmacist	9	5%
Radiation oncologist	1	1%
Other	1	1%
In total, how many years have you been taking care of patients with lymphoma? (*N* = 167)		
Median	10	
Range	1.5–40	
Approximately how many patients with DLBCL do you see per week? (*N* = 167)		
Median	5	
Range	0–70	
What is the extent of your participation in clinical trials specifically enrolling patients with DLBCL? Select all that apply (*N* = 174)		
PI	75	43%
Coinvestigator	80	46%
Local site PI	79	45%
Enroll patients onto clinical trials	85	49%
None of the above	27	16%
What is your preferred strategy for treating patients with newly diagnosed *early‐stage* (Ann‐Arbor I–II, non‐bulky) DLBCL? (*N* = 166)		
R‐CHOP × 4 cycles	63	38%
R‐CHOP × 3 cycles followed by ISRT	58	35%
R‐CHOP × 6 cycles ± ISRT	27	16%
Other	18	11%
How do you generally treat newly diagnosed *advanced‐stage* (Ann Arbor III–IV) *double expressor* (negative for MYC rearrangement) DLBCL? (*N* = 165)		
R‐CHOP × 6 cycles	146	88%
Dose‐adjusted R‐EPOCH × 6 cycles	13	8%
Other	6	4%
How do you generally treat newly diagnosed *advanced‐stage* high‐grade B‐cell lymphoma with MYC and BCL2 and/or BCL6 rearrangements (also known as **“** *double‐hit*” or **“** *triple‐hit*” lymphoma)? (*N* = 166)		
R‐CHOP × 6 cycles	39	23%
Dose‐adjusted R‐EPOCH × 6 cycles	111	67%
Other	16	10%
Do you use CNS prophylaxis in patients with DLBCL and a high CNS‐IPI score (i.e., several extranodal sites, kidney/adrenal involvement), and if so, what is your preferred strategy? (*N* = 165)		
Yes, and I prefer IT methotrexate	28	17%
Yes, and I prefer IV high‐dose methotrexate	78	47%
Yes, but I have no inherent preference between IT and IV methotrexate	15	9%
No, I do not routinely consider CNS prophylaxis in this setting	35	21%
Other	9	5%
What is your clinical experience so far treating patients with relapsed or refractory DLBCL with polatuzumab vedotin? (*N* = 166)		
I have had generally good outcomes	36	22%
I have had a mix of good and poor outcomes	80	48%
I have had generally poor outcomes	20	12%
I have not used polatuzumab vedotin in the relapsed or refractory setting	27	16%
Other	3	2%

Abbreviations: CNS, central nervous system; IPI, International Prognostic Index; ISRT, involved‐site radiation therapy; IT, intrathecal; IV, intravenous; PI, principal investigator.

When presented with a hypothetical new frontline DLBCL regimen “S‐FLOP” with a 15% absolute improvement in 2‐year PFS but no difference in OS compared to R‐CHOP, most respondents chose “S‐FLOP” over R‐CHOP (78%). Respondents ranked OS as the most important consideration in adopting “S‐FLOP,” followed by PFS and adverse events. Additional costs of “S‐FLOP,” patient‐reported outcomes, and subsequent therapies were ranked as less important. If “S‐FLOP” was twice as expensive as R‐CHOP, 52% recommended “S‐FLOP” over R‐CHOP. When the PFS benefit changed to 5%, with twice the cost, only 20% recommended “S‐FLOP.”

In response to the PFS and OS results from POLARIX, 30% of respondents stated they would replace R‐CHOP with pola‐R‐CHP (Table 2). In the analysis of the free‐text responses, respondents who recommended pola‐R‐CHP stated that the PFS benefit was important, whereas those who did not recommend pola‐R‐CHP highlighted an insufficient PFS difference, lack of OS benefit demonstrated to date, and excessive costs. Sixty percent of respondents felt that pola‐R‐CHP and R‐CHOP had similar toxicity profiles, whereas 36% felt that pola‐R‐CHP was mildly more toxic. Many respondents did not plan to use pola‐R‐CHP regardless of the toxicity profile (50%) due to underwhelming efficacy.

**TABLE 2:** Impressions about the results of the POLARIX study by survey respondents

Survey question	Total (*N* = 174)	%
Are you familiar with the results of the POLARIX study? (*N* = 137)		
Yes	131	96%
No	6	4%
Do you plan to replace R‐CHOP with pola‐R‐CHP in your practice for patients with newly diagnosed DLBCL? (*N* = 145)		
Yes	43	30%
No	102	70%
Does information about overall response and complete response rates change your previously expressed impression of pola‐R‐CHP compared to R‐CHOP? (*N* = 145)		
Yes	6	4%
No	139	96%
With this information about safety and toxicities in the POLARIX study, how do you view the toxicity profiles of pola‐R‐CHP and R‐CHOP? (*N* = 144)		
Pola‐R‐CHP is significantly more toxic than R‐CHOP	2	1%
Pola‐R‐CHP is mildly more toxic than R‐CHOP	52	36%
Pola‐R‐CHP and R‐CHOP have similar toxicity profiles	87	60%
R‐CHOP is mildly more toxic than pola‐R‐CHP	3	2%
R‐CHOP is significantly more toxic than pola‐R‐CHP	0	0%
Which of the following statements best describes how the toxicity profile of pola‐R‐CHP influences your thoughts on utilizing this regimen instead of R‐CHOP? (*N* = 145)		
The toxicity profile of pola‐R‐CHP makes me more likely to utilize it in place of R‐CHOP	24	17%
Irrespective of the toxicity profile of pola‐R‐CHP, I plan to utilize it in place of R‐CHOP due to impressive efficacy outcomes	17	12%
Irrespective of the toxicity profile of pola‐R‐CHP, I do not plan to utilize it in place of R‐CHOP due to underwhelming efficacy outcomes	73	50%
The toxicity profile of pola‐R‐CHP makes me less likely to utilize it in place of R‐CHOP	19	13%
Other	12	8%
Based on your impression of the information presented thus far, what would be a fair cost for one cycle of pola‐R‐CHP? (*N* = 146)		
Greater than $20,000 USD	10	7%
$15,000–$20,000 USD	11	8%
$10,000–$15,000 USD	38	26%
$8000–$10,000 USD	56	38%
$7000 USD (the same as R‐CHOP)	27	18%
Other	4	3%
Based on the information presented thus far about the cost of one cycle of pola‐R‐CHP, do you plan to replace R‐CHOP with pola‐R‐CHP in your practice for patients with newly diagnosed DLBCL? (*N* = 140)		
Yes	32	23%
No	108	77%
How do the financial implications of offering pola‐R‐CHP to your patients with DLBCL influence your decision to offer it over R‐CHOP? (*N* = 140)		
A. I definitely will offer pola‐R‐CHP irrespective of the financial implications	22	16%
B. I am hesitant to offer pola‐R‐CHP due to financial implications for society and my country's healthcare system at‐large	33	24%
C. I am hesitant to offer pola‐R‐CHP due to financial implications for my patients specifically	11	8%
Both B and C	29	21%
D. I am hesitant to offer pola‐R‐CHP irrespective of the financial implications because of concerns about the POLARIX trial design, endpoints, and/or outcomes	29	21%
E. None of the above apply to me	16	11%
Does this information regarding exploratory subgroup analyses influence your previously expressed opinions? (*N* = 140)		
Yes	62	44%
No	78	56%
Before considering a change in your typical management of patients with newly diagnosed advanced‐stage DLBCL, which of the following options is closest to your desired minimum NNT with a regimen other than R‐CHOP to achieve one additional cure with frontline therapy? (*N* = 139)		
5	14	10%
10	32	23%
15	18	13%
20	12	9%
30	3	2%
I do not routinely think about NNT in this context	57	41%
Other	3	2%
In POLARIX, approximately 17 patients needed to receive pola‐R‐CHP to cure one additional patient with frontline therapy, at an approximate cost of $1.6 million USD to the healthcare system. The NNT to avoid one additional patient having to undergo autologous stem‐cell transplantation was 32 (approximately $3.1 million), and to avoid one additional patient having to undergo CAR T‐cell therapy was 63 (approximately $6 million, based on CAR T use in the third‐line setting). How does this information influence your views? (*N* = 137)		
I am much more likely to offer pola‐R‐CHP over R‐CHOP	6	4%
I am somewhat more likely to offer pola‐R‐CHP over R‐CHOP	19	14%
My views are not influenced by these data	64	47%
I am somewhat less likely to offer pola‐R‐CHP over R‐CHOP	31	23%
I am much less likely to offer pola‐R‐CHP over R‐CHOP	17	12%

Abbreviations: CAR, chimeric antigen receptor; DLBCL, diffuse large B‐cell lymphoma; NNT, number needed to treat.

When presented with approximate costs‐per‐cycle of pola‐R‐CHP, 23% of all respondents indicated they would replace R‐CHOP with pola‐R‐CHP (Table 2). Free‐text responses revealed that these respondents felt that PFS benefits superseded cost concerns and that physicians are not responsible for determining acceptable costs, whereas those who did not recommend pola‐R‐CHP felt that the PFS benefit was underwhelming relative to the excessive cost and that the regimen would not be available owing to lack of reimbursement or approval. Most commonly, respondents were hesitant to offer pola‐R‐CHP due to financial implications for society (24%) or financial implications for society and patients (29%).

Regarding exploratory subgroup analyses of POLARIX demonstrating PFS benefit in patients with activated B‐cell (ABC) DLBCL, 56% of respondents stated that this information did not influence their previous opinions about pola‐R‐CHP; many felt that definitive decisions could not be made based on underpowered subgroup analyses. Many respondents did not routinely use the number needed to treat (NNT) for PFS in decision‐making about frontline DLBCL management (41%), and their opinions regarding pola‐R‐CHP were not influenced when presented with NNT data for pola‐R‐CHP (47%).

Unlike other malignancies [6, 7], PFS substantially reflects the curative potential of initial DLBCL treatment, with strong trial‐level surrogacy of PFS for OS [8]. Despite its statistical significance, the PFS improvement with pola‐R‐CHP (hazard ratio, 0.73; 2‐year PFS 76.7% vs. 70.2%) was viewed by most respondents as underwhelming. When presented with pola‐R‐CHP costs, support for pola‐R‐CHP declined further. To assess for bias against pola‐R‐CHP, respondents were first confronted with a DCE involving a hypothetical “S‐FLOP” regimen compared to R‐CHOP. Most respondents demonstrated internal consistency, indicating declining support for “S‐FLOP” over R‐CHOP when the PFS benefit shrank and/or costs increased.

This survey reveals several open questions regarding the future of frontline DLBCL management. First, 70% of respondents would not yet replace R‐CHOP with pola‐R‐CHP, with a modest PFS benefit and costs being primary reasons. However, based on the DCE findings, there may be an absolute PFS difference that clinicians would accept despite higher costs. Second, although most respondents do not explicitly consider NNT, the majority expressed concern about societal financial implications of pola‐R‐CHP, suggesting an alternative calculation of “cost‐needed‐to‐cure” when weighing costs of new DLBCL treatments against PFS improvements. Finally, varying international interpretations of the implications of POLARIX data, as highlighted in our study, suggest that practice patterns are likely to diverge globally, complicating the development of a new standard‐of‐care.

The primary limitation of this study is that most respondents were academic clinicians with clinical trial experience who were already aware of the POLARIX study results, which limited the capture of opinions of nonacademic clinicians. Additionally, our survey collected impressions of the initial publication of POLARIX study results, but data from longer follow‐up may reveal new insights, especially when the primary OS analysis is completed. Study strengths include a significant proportion of respondents outside of the US and the timing of our survey, which was distributed prior to pola‐R‐CHP entering society guidelines and routine practice.

The observed heterogeneity in POLARIX interpretation suggests that, in the real‐world setting, clinicians may use pola‐R‐CHP in (1) all patients meeting POLARIX eligibility criteria (i.e., IPI score ≥2), (2) only specific subgroups of patients (e.g., ABC subtype), or (3) no patients unless a future OS benefit is demonstrated or the price of polatuzumab changes, among other views. With many respondents stating that the PFS benefit from pola‐R‐CHP is practice‐changing regardless of costs, future formal cost‐effectiveness, and clinician practice pattern studies are warranted, given ongoing frontline DLBCL studies with R‐CHOP control arms.

## CONFLICT OF INTEREST

The authors declare they have no conflicts of interest.

## FUNDING INFORMATION

The authors received no specific funding for this work.

## ETHICS STATEMENT

This study was approved with exempt status by Washington University in St. Louis Human Research Protection Office, IRB 202201013.

## AUTHOR CONTRIBUTIONS

Ajay Major and David Russler‐Germain conceived the study. Ajay Major, Edward R. Scheffer Cliff, Daniel A. Ermann, and David A. Russler‐Germain developed the electronic survey instrument, analyzed the results, and wrote the manuscript.

## DATA AVAILABILTY STATEMENT

Anonymized survey response areas are available to qualified investigators via email request to the corresponding author.

## Supporting information

Supplement Material
