# Supplementary material for: Frontline polatuzumab vedotin for diffuse large B‐cell lymphoma: A survey of clinician impressions
Source: EJHaem. 2022 Jun 16;3(3):930–5. doi: 10.1002/jha2.505 (PMC9422021; doi:10.1002/jha2.505)
Supplement: Supplementary file 1 — Supplement Material [file JHA2-3-930-s001.pdf]

A Survey of Lymphoma Clinician Impressions

Dear colleague,

You are invited to participate in an academic research study entitled, “A Survey of Lymphoma Clinician Impressions.” This study is being conducted by Dr. David Russler-Germain from Washington University in St. Louis and Dr. Ajay Major from the University of Chicago. You were selected to participate in this study because you treat patients with lymphoma.

The purpose of this study is to understand practice patterns of lymphoma clinicians and attitudes towards polatuzumab vedotin (PolivyTM; Genentech) in the management of diffuse large B-cell lymphoma (DLBCL). If you agree to participate in this study, you will be asked to complete an online survey which takes 10 minutes to complete.

You may not benefit directly from this research; however, we hope that your participation in the study may help the lymphoma community at-large understand how polatuzumab vedotin is and might soon be used in ‘real-world’ practice.

We believe there are no known risks associated with this research study. To the best of our ability, your responses will remain anonymous and confidential on a secure server. The writing of the survey questions and the analysis of the responses have been and will be free from influence by any pharmaceutical industry parties. Your participation in this study is completely voluntary and you can withdraw consent at any time.

If you have any questions or concerns about the project, you may contact Dr. Ajay Major at ajay.major@uchospitals.edu and Dr. David Russler-Germain at germaind@wustl.edu. If you are aware of other individuals or groups not yet invited to participate in this survey but who you would recommend this survey to, please inform Drs. Major and Russler-Germain via email at your convenience.

This study was approved with exempt status by the Washington University in St. Louis Human Research Protection Office, IRB #202201013.

By clicking "I agree," you are indicating that you are a clinician, are at least 18 years old, have read and understood this consent form, and agree to participate in this research study.	◯ I agree<br>◯ I do not agree
Which best describes the healthcare setting in which you work?	◯ Academic health system<br>◯ Private practice<br>◯ Hybrid model (private with academic affiliation)<br>◯ Other
If you selected 'other,' please describe your practice setting.	__________________________________

In what country are you located?

◯ Afghanistan
◯ Albania
◯ Algeria
◯ Andorra
◯ Angola
◯ Antigua and Barbuda
◯ Argentina
◯ Armenia
◯ Australia
◯ Austria
◯ Azerbaijan
◯ Bahamas
◯ Bahrain
◯ Bangladesh
◯ Barbados
◯ Belarus
◯ Belgium
◯ Belize
◯ Benin
◯ Bhutan
◯ Bolivia
◯ Bosnia and Herzegovina
◯ Botswana
◯ Brazil
◯ Brunei
◯ Bulgaria
◯ Burkina Faso
◯ Burundi
◯ Cabo Verde
◯ Cambodia
◯ Cameroon
◯ Canada
◯ Central African Republic (CAR)
◯ Chad
◯ Chile
◯ China
◯ Colombia
◯ Comoros
◯ Democratic Republic of the Congo
◯ Republic of the Congo
◯ Costa Rica
◯ Cote d'Ivoire
◯ Croatia
◯ Cuba
◯ Cyprus
◯ Czechia
◯ Denmark
◯ Djibouti
◯ Dominica
◯ Dominican Republic
◯ Ecuador
◯ Egypt
◯ El Salvador
◯ Equatorial Guinea
◯ Eritrea
◯ Estonia
◯ Eswatini
◯ Ethiopia
◯ Fiji
◯ Finland
◯ France
◯ Gabon
◯ Gambia
◯ Georgia
◯ Germany
◯ Ghana
◯ Greece
◯ Grenada
◯ Guatemala

◯ Guinea
◯ Guinea-Bissau
◯ Guyana
◯ Haiti
◯ Honduras
◯ Hungary
◯ Iceland
◯ India
◯ Indonesia
◯ Iran
◯ Iraq
◯ Ireland
◯ Israel
◯ Italy
◯ Jamaica
◯ Japan
◯ Jordan
◯ Kazakhstan
◯ Kenya
◯ Kiribati
◯ Kosovo
◯ Kuwait
◯ Kyrgyzstan
◯ Laos
◯ Latvia
◯ Lebanon
◯ Lesotho
◯ Liberia
◯ Libya
◯ Liechtenstein
◯ Lithuania
◯ Luxembourg
◯ Madagascar
◯ Malawi
◯ Malaysia
◯ Maldives
◯ Mali
◯ Malta
◯ Marshall Islands
◯ Mauritania
◯ Mauritius
◯ Mexico
◯ Micronesia
◯ Moldova
◯ Monaco
◯ Mongolia
◯ Montenegro
◯ Morocco
◯ Mozambique
◯ Myanmar
◯ Namibia
◯ Nauru
◯ Nepal
◯ Netherlands
◯ New Zealand
◯ Nicaragua
◯ Niger
◯ Nigeria
◯ North Korea
◯ North Macedonia
◯ Norway
◯ Oman
◯ Pakistan
◯ Palau
◯ Palestine
◯ Panama
◯ Papua New Guinea
◯ Paraguay
◯ Peru
◯ Philippines
◯ Poland

	◯ Portugal<br>◯ Qatar<br>◯ Romania<br>◯ Russia<br>◯ Rwanda<br>◯ Saint Kitts and Nevis<br>◯ Saint Lucia<br>◯ Saint Vincent and the Grenadines<br>◯ Samoa<br>◯ San Marino<br>◯ Sao Tome and Principe<br>◯ Saudi Arabia<br>◯ Senegal<br>◯ Serbia<br>◯ Seychelles<br>◯ Sierra Leone<br>◯ Singapore<br>◯ Slovakia<br>◯ Slovenia<br>◯ Solomon Islands<br>◯ Somalia<br>◯ South Africa<br>◯ South Korea<br>◯ South Sudan<br>◯ Spain<br>◯ Sri Lanka<br>◯ Sudan<br>◯ Suriname<br>◯ Sweden<br>◯ Switzerland<br>◯ Syria<br>◯ Taiwan<br>◯ Tajikistan<br>◯ Tanzania<br>◯ Thailand<br>◯ Timor-Leste<br>◯ Togo<br>◯ Tonga<br>◯ Trinidad and Tobago<br>◯ Tunisia<br>◯ Turkey<br>◯ Turkmenistan<br>◯ Tuvalu<br>◯ Uganda<br>◯ Ukraine<br>◯ United Arab Emirates (UAE)<br>◯ United Kingdom (UK)<br>◯ United States of America (USA)<br>◯ Uruguay<br>◯ Uzbekistan<br>◯ Vanuatu<br>◯ Vatican City (Holy See)<br>◯ Venezuela<br>◯ Vietnam<br>◯ Yemen<br>◯ Zambia<br>◯ Zimbabwe
What is your degree?	◯ MD, DO, MBBS, MBChB, or equivalent<br>◯ MD + PhD<br>◯ PA<br>◯ NP (i.e., APN, DNP, or equivalent)<br>◯ PharmD or other pharmacy degree<br>◯ Other

If you selected 'other,' please describe your degree.	__________________________________
What type of clinician are you?	◯ Hematologist<br>◯ Medical oncologist<br>◯ Hematologist / medical oncologist<br>◯ Radiation oncologist<br>◯ Hematology, oncology, or hematology/oncology fellow<br>◯ Pharmacist<br>◯ Other
If you selected 'other,' please describe what type of clinician you are.	__________________________________
In total, how many years have you been taking care of patients with lymphoma?	__________________________________
Approximately how many patients with DLBCL do you see per week?	__________________________________
What is the extent of your participation in clinical trials specifically enrolling patients with DLBCL? Select all that apply.	☐ Principal investigator (PI)<br>☐ Co-investigator<br>☐ Local site PI<br>☐ Enroll patients onto clinical trials<br>☐ None of the above
What is your preferred strategy for treating patients with newly-diagnosed early-stage (Ann-Arbor I-II, non-bulky) DLBCL?	◯ R-CHOP x3 cycles followed by ISRT<br>◯ R-CHOP x6 cycles ± ISRT<br>◯ R-CHOP x4 cycles ± 2 additional cycles of rituximab monotherapy<br>◯ Other
If you selected 'other,' please describe your treatment strategy for newly-diagnosed early-stage DLBCL.	__________________________________
How do you generally treat newly-diagnosed advanced-stage (Ann Arbor III–IV) double expressor (negative for MYC rearrangement) DLBCL?	◯ R-CHOP x6 cycles<br>◯ Dose-adjusted R-EPOCH x6 cycles<br>◯ Other
If you selected 'other,' please describe your treatment strategy for newly-diagnosed advanced-stage double expressor DLBCL.	__________________________________
How do you generally treat newly-diagnosed advanced-stage high-grade B-cell lymphoma with MYC and BCL2 and/or BCL6 rearrangements (also known as ‘double-hit’ or ‘triple-hit’ lymphoma)?	◯ R-CHOP x6 cycles<br>◯ Dose-adjusted R-EPOCH x6 cycles<br>◯ Other
If you selected 'other,' please describe your treatment strategy for newly-diagnosed advanced-stage high-grade B-cell lymphoma with MYC and BCL2 and/or BCL6 rearrangements.	__________________________________

Do you use central nervous system (CNS) prophylaxis in patients with DLBCL and a high CNS-IPI score (i.e., several extranodal sites, kidney/adrenal involvement), and if so, what is your preferred strategy?	◯ Yes, and I prefer intrathecal (IT) methotrexate<br>◯ Yes, and I prefer intravenous (IV) high-dose methotrexate<br>◯ Yes, but I have no inherent preference between IT and IV methotrexate<br>◯ No, I do not routinely consider CNS prophylaxis in this setting<br>◯ Other
If you selected 'other,' please describe your CNS prophylaxis strategy in this setting.	__________________________________
What is your clinical experience so far treating patients with relapsed or refractory DLBCL with polatuzumab vedotin?	◯ I have had generally good outcomes<br>◯ I have had a mix of good and poor outcomes<br>◯ I have had generally poor outcomes<br>◯ I have not used polatuzumab vedotin in the relapsed or refractory setting<br>◯ Other
If you selected 'other,' please describe your clinical experience treating patients with relapsed or refractory DLBCL with polatuzumab vedotin.	__________________________________

R-CHOP has been the standard of care for the management of newly-diagnosed DLBCL for over a decade, with numerous clinical trials being unsuccessful in improving outcomes.

In these next questions, we propose a hypothetical new regimen "S-FLOP" that is studied in a phase 3 randomized controlled trial (RCT) against R-CHOP in patients with newly-diagnosed advanced-stage DLBCL. The specific agents in S-FLOP are not specified to avoid biases related to potential attractiveness of their mechanisms of action, novelty, and/or relevance to the treatment of relapsed DLBCL.

In this hypothetical RCT of R-CHOP versus S-FLOP, a 15% absolute improvement in progression-free survival (PFS) at 24-months is demonstrated with S-FLOP, with no difference in overall survival (OS).

Please rank the following items from most (#1) to least (#7) important to you when considering adoption of the hypothetical S-FLOP regimen above for your patients with newly-diagnosed DLBCL.

	1 (most important)	2	3	4	5	6	7 (least important)
Progression-free survival	◯	◯	◯	◯	◯	◯	◯
Complete response rate	◯	◯	◯	◯	◯	◯	◯
Overall survival	◯	◯	◯	◯	◯	◯	◯
Adverse events / side effects between the two regimens	◯	◯	◯	◯	◯	◯	◯
Additional cost of S-FLOP regimen	◯	◯	◯	◯	◯	◯	◯
Patient-reported outcomes	◯	◯	◯	◯	◯	◯	◯
Subsequent therapies after either regimen	◯	◯	◯	◯	◯	◯	◯

Given a 15% PFS benefit with S-FLOP over R-CHOP without a difference in OS, which would you recommend to your patients, ignoring toxicity or cost?	◯ S-FLOP ◯ R-CHOP

Treating a patient with the hypothetical S-FLOP regimen costs twice as much overall compared to R-CHOP, with similar toxicity. Knowing this, which regimen would you recommend for your patients?	◯ S-FLOP ◯ R-CHOP
If instead the PFS benefit of S-FLOP was only 5% greater than R-CHOP, and the cost remained twice as much overall compared to R-CHOP, which regimen would you recommend to your patients?	◯ S-FLOP ◯ R-CHOP
Are you familiar with the results of the POLARIX study, which were presented at the American Society of Hematology (ASH) 2021 Annual Meeting and published in The New England Journal of Medicine on December 14, 2021?<br><br>Please email Dr. Ajay Major at ajay.major@uchospitals.edu and/or Dr. David Russler-Germain at germaind@wustl.edu if you do not have access to this paper.	◯ Yes<br>◯ No

The POLARIX study is a double-blind, placebo-controlled, international phase 3 RCT evaluating a modified regimen of R-CHOP (pola-R-CHP), in which vincristine was replaced with polatuzumab vedotin, compared with standard R-CHOP, in patients with previously untreated intermediate- or high-risk (international prognostic index [IPI] ≥2) DLBCL of any stage. Patients were 18–80 years of age and received six cycles of either pola-R-CHP or R-CHOP, plus two additional cycles of rituximab alone.

The primary endpoint was investigator-assessed progression-free survival (PFS), and secondary endpoints included overall survival (OS) and safety. In total, 879 patients were randomized and median follow-up at time of publication was 28.2 months. Regarding efficacy, PFS at 24-months was significantly higher in the pola-R-CHP group than the R-CHOP group (76.7% vs. 70.2%, respectively; hazard ratio [HR] = 0.73, 95% confidence interval [CI] = 0.57–0.95, P = 0.02). Overall survival at 24-months was not significantly different (88.7% vs. 88.6%, respectively; HR = 0.94, 95% CI = 0.65–1.37, P = 0.75).

Based on the information presented above, do you plan to replace R-CHOP with pola-R-CHP in your practice for patients with newly-diagnosed DLBCL?	◯ Yes<br>◯ No
If yes, why?	__________________________
If no, why not?	__________________________

In POLARIX, response rates were comparable in the two arms of the study. Overall response rates were 85.5% and 83.8% in the pola-R-CHP and R-CHOP groups, respectively, and complete response rates were 78.0% and 74.0%, respectively. Neither difference was statistically significant.

Does this additional information change your previously expressed impression of pola-R-CHP compared to R-CHOP?	◯ Yes<br>◯ No
If yes, why?	__________________________
If no, why not?	__________________________

In POLARIX, the overall safety profile of pola-R-CHP was described as 'generally similar' compared to R-CHOP, given 'mostly similar types and incidences' of adverse events (AEs). The most common AEs of grade 3+ were neutropenia (28.3% in pola-R-CHP group vs. 30.8% in R-CHOP group), febrile neutropenia (13.8% vs. 8.0%, respectively), and anemia (12.0% vs. 8.4%, respectively). The percentages of patients who had infections of grade 3+ were 'similar' (15.2% in pola-R-CHP group vs. 12.6% in R-CHOP group), as were the percentages of patients who discontinued at least one drug in the regimen (2.1% vs. 2.3%, respectively) or had dose reductions (1.8% vs. 2.5%, respectively) because of either infections or neutropenia. Grade 5 AEs (i.e., resulting in death) were primarily related to infections and occurred in 13 patients in the pola-R-CHP group and in 10 patients in the R-CHOP group. Peripheral neuropathy of any grade was reported in 52.9% of patients receiving pola-R-CHP and in 53.9% of patients receiving R-CHOP.

With this additional information, how do you view the toxicity profiles of pola-R-CHP and R-CHOP?

- ◯ Pola-R-CHP is significantly more toxic than R-CHOP
- ◯ Pola-R-CHP is mildly more toxic than R-CHOP
- ◯ Pola-R-CHP and R-CHOP have similar toxicity profiles
- ◯ R-CHOP is mildly more toxic than pola-R-CHP
- ◯ R-CHOP is significantly more toxic than pola-R-CHP

Which of the following statements best describes how the toxicity profile of pola-R-CHP influences your thoughts on utilizing this regimen instead of R-CHOP?

- ◯ The toxicity profile of pola-R-CHP makes me more likely to utilize it in place of R-CHOP
- ◯ Irrespective of the toxicity profile of pola-R-CHP, I plan to utilize it in place of R-CHOP due to impressive efficacy outcomes
- ◯ Irrespective of the toxicity profile of pola-R-CHP, I do not plan to utilize it in place of R-CHOP due to underwhelming efficacy outcomes
- ◯ The toxicity profile of pola-R-CHP makes me less likely to utilize it in place of R-CHOP
- ◯ Other

If you selected 'other,' please elaborate how the toxicity profile of pola-R-CHP influences your thoughts on utilizing this regimen instead of R-CHOP.

____

As of the date of publication of the POLARIX study, the approximate cost of one cycle of standard-of-care R-CHOP for a patient in the United States of America (USA) was $7,000 USD using the least expensive published average wholesale prices of the included medications, not including any facility and professional fees.

Based on your impression of the information presented thus far, what would be a fair cost for one cycle of pola-R-CHP?

- ◯ Greater than $20,000 USD
- ◯ $15,000-$20,000 USD
- ◯ $10,000-$15,000 USD
- ◯ $8,000-$10,000 USD
- ◯ $7,000 USD (the same as R-CHOP)
- ◯ Other

If you selected 'other,' please state your fair cost for one cycle of pola-R-CHP.

____

Based on the average wholesale price of polatuzumab vedotin in the USA as of the date of publication of the POLARIX study, it is estimated that one cycle of pola-R-CHP will cost approximately $23,000 USD, not including any facility and professional fees and also not including granulocyte colony-stimulating factor (GCSF) required for patients of all ages per POLARIX (in contrast to the typical use of GCSF for primary prophylaxis against neutropenia with R-CHOP only in patients over the age of 60 years).

Based on the information presented thus far, do you plan to replace R-CHOP with pola-R-CHP in your practice for patients with newly-diagnosed DLBCL?

- ◯ Yes
- ◯ No

If yes, why?

____

If no, why not?

____

How do the financial implications of offering pola-R-CHP to your patients with DLBCL influence your decision to offer it over R-CHOP?

- ◯ A. I definitely will offer pola-R-CHP irrespective of the financial implications
- ◯ B. I am hesitant to offer pola-R-CHP due to financial implications for society and my country's healthcare system at-large
- ◯ C. I am hesitant to offer pola-R-CHP due to financial implications for my patients specifically
- ◯ Both B and C
- ◯ D. I am hesitant to offer pola-R-CHP irrespective of the financial implications because of concerns about the POLARIX trial design, endpoints, and/or outcomes
- ◯ E. None of the above apply to me

One of the largest differences in treatment effect for the exploratory subgroup analysis (i.e., not tested for statistical significance) from the POLARIX study was the benefit seen in patients with activated B-cell (ABC) cell-of-origin subtype DLBCL, with PFS HR 0.4 (95% CI 0.2-0.6) and 24-month PFS of 83.9% for pola-R-CHP (n = 102) versus 58.8% for R-CHOP (n = 119).

Does this additional information influence your previously expressed opinions?

- ◯ Yes
- ◯ No

If yes, why?

____

If no, why not?

____

Before considering a change in your typical management of patients with newly-diagnosed advanced-stage DLBCL, which of the following options is closest to your desired minimum number needed to treat (NNT) with a regimen other than R-CHOP to achieve one additional cure with frontline therapy?

- ◯ 5
- ◯ 10
- ◯ 15
- ◯ 20
- ◯ 30
- ◯ I do not routinely think about NNT in this context
- ◯ Other

If you selected 'other,' please elaborate.

____

In POLARIX, 17 patients needed to receive pola-R-CHP to cure one additional patient with frontline therapy, at an approximate cost of $1.6 million USD to the healthcare system. The number needed to treat (NNT) to avoid one additional patient having to undergo autologous stem cell transplantation was 32 (approximately $3.1 million), and to avoid one additional patient having to undergo CAR T-cell therapy was 63 (approximately $6 million, based on CAR T use in the third-line setting).

How does this information influence your views?

- ◯ I am much more likely to offer pola-R-CHP over R-CHOP
- ◯ I am somewhat more likely to offer pola-R-CHP over R-CHOP
- ◯ My views aren't influenced by these data
- ◯ I am somewhat less likely to offer pola-R-CHP over R-CHOP
- ◯ I am much less likely to offer pola-R-CHP over R-CHOP

Are there additional opinions you have regarding the POLARIX trial or polatuzumab vedotin in general that you feel would be informative for this study? ____
